# AI-powered rapid detection of multidrug-resistant *Klebsiella pneumoniae* with informative peaks of MALDI-TOF MS

**DOI:** 10.1093/bioadv/vbaf303

**Published:** 2025-11-24

**Authors:** Jang-Jih Lu, Chia-Ru Chung, Hsin-Yao Wang, Yun Tang, Ming-Chien Chiang, Li-Ching Wu, Justin Bo-Kai Hsu, Tzong-Yi Lee, Jorng-Tzong Horng

**Affiliations:** Division of Clinical Pathology, Taipei Tzu Chi Hospital, Buddhist Tzu Chi Medical Foundation, New Taipei City, 231016, Taiwan; Department of Computer Science and Information Engineering, National Central University, Taoyuan, 320317, Taiwan; Bruker, Billerica, MA, 01821, United States; Institute of Bioinformatics and Systems Biology, National Yang Ming Chiao Tung University, Hsinchu, 300193, Taiwan; Department of Computer Science and Information Engineering, National Central University, Taoyuan, 320317, Taiwan; Department of Biomedical Sciences and Engineering, National Central University, Taoyuan, 320317, Taiwan; Department of Computer Science and Engineering, Yuan Ze University, Taoyuan, 320315, Taiwan; Institute of Bioinformatics and Systems Biology, National Yang Ming Chiao Tung University, Hsinchu, 300193, Taiwan; Center for Intelligent Drug Systems and Smart Biodevices (IDS2B), National Yang Ming Chiao Tung University, Hsinchu City, 300193, Taiwan; Department of Computer Science and Information Engineering, National Central University, Taoyuan, 320317, Taiwan

## Abstract

**Motivation:**

*Klebsiella pneumoniae* is a highly virulent superbug with rising antibiotic resistance worldwide. While matrix-assisted laser desorption/ionization time-of-flight mass spectrometry (MALDI-TOF MS) has transformed microbial identification, its application to antimicrobial resistance prediction remains underexplored, particularly for large clinical cohorts. In this study, we developed machine-learning models with feature-level interpretability using MALDI-TOF MS data to rapidly predict resistance to ciprofloxacin (CIP), cefuroxime (CXM), and ceftriaxone (CRO) in *K. pneumoniae*.

**Results:**

Using more than 28 000 isolates from two hospitals, the best-performing models reached an independent test accuracy of 0.7858, with sensitivity of 0.7289 and specificity of 0.8127. Several resistance-associated *m/z* signals—including 3657, 4341, 4519, 4709, 5070, 5409, 5921, 5939, and 6516—were consistently enriched in resistant isolates, offering interpretable spectral markers linked to resistance. Performance remained stable in time-based validation but declined across hospitals, suggesting sensitivity to geographic variability in resistance profiles. Overall, this study demonstrates that combining MALDI-TOF MS with machine learning enables rapid and interpretable prediction of resistance to commonly used fluoroquinolone and cephalosporins in *K. pneumoniae*. These findings highlight the clinical potential of such models for supporting empiric therapy and emphasize the importance of incorporating local data or adaptive strategies to improve generalizability across healthcare settings.

**Availability and implementation:**

Data available on request from the authors.

## 1 Introduction

*Klebsiella pneumoniae*, a Gram-negative bacterium, can cause a variety of serious infections (Ko *et al.* 2002). These infections are often treated with antibiotics, but overusing and misusing these drugs can lead to the development of antibiotic-resistant *K. pneumoniae* (Gasink *et al.* 2009, Kim *et al.* 2016). This study focused on three antibiotics commonly used to treat *K. pneumoniae* infections: ciprofloxacin (CIP), cefuroxime (CRO), and ceftriaxone (CXM). These drugs work by binding to different proteins in the bacterial cell, inhibiting their growth and replication. Detailed information is provided in Table 1, available as supplementary data at *Bioinformatics Advances* online (Rawat and Nair 2010, Kim *et al.* 2019). However, the emergence of antibiotic-resistant *K. pneumoniae* is a significant problem in clinical treatment, particularly when the bacteria produce extended-spectrum β-lactamases (ESBLs), which can confer resistance to multiple antibiotics (Wang *et al.* 2004, Paterson and Bonomo 2005). To address this issue, an accurate antibiotic susceptibility test (AST) is critical, but current methodologies often require up to 24 h to yield results (Khan *et al.* 2019).

**Table 1.** Summary of studies utilizing MALDI-TOF MS to investigate antibiotic resistance of *K. pneumoniae*.^a^

Antibiotics/phenotype	Data (R/S); setting	Performance (as reported)	#Feature (tools)	Informative peaks/notes	References
CRKP	25 (25/0); single center	AUROC ranged from 0.85 to 1.00	Peak-based (ClinProTools)	Discriminatory peaks observed at *m/z* 4154, 4738, 4770, 5381, 6096, 6152, 6289, 7244, 8308, and 9476.	Angeletti *et al.* (2015)
Carbapenem- and colistin-resistant *K. pneumoniae*	36 (16/20); single center	AUROC 0.86; SN 93.8%; SP 55.0%	Peak-based (ClinProTools)	The signal at *m/z* 6100 was absent in most resistant isolates.	Flores-Treviño *et al.* (2019)
Carbapenemase (KPC) detection	373 (210/163); multi-laboratory	SN 95.7%; SP 100%	Peak-based (VITEK MS RUO subtyping)	The signal at *m/z* 11 109, corresponding to a plasmid protein (p019), was detected in most KPC producers.	Rocco *et al.* (2019)
CRKP versus CSKP	95 (46/49); single center	SN 93%; SP 100%; ACC 97%	80 peaks (RF)	Discriminatory peaks observed at *m/z* 2636.880, 4362.217, 4768.279, 5379.418, 6288.794, 7158.634, 7705.009, 9541.405, 9478.866, and 10 287.76.	Huang *et al.* (2020)
KPC detection (methods review)	Not applicable	Not applicable	Not applicable	Reviewed methodological approaches; highlighted the diagnostic role of the signal at *m/z* 11 109 and β-lactam hydrolysis peaks.	Hleba *et al.* (2021)
CRKP versus CSKP	171 (95/76); single center	AUROC 0.936; ACC 0.91; SN 0.89; SP 0.94	Engineered spectral features (RF, SVM)	Discriminatory features included a peak at *m/z* ∼6515 and additional signals identified by ML models.	Wang *et al.* (2022)
Multiple antibiotics including *K. pneumoniae*–CIP, *K. pneumoniae*–CRO	More than 300 000 spectra; multi-center	AUROC ≈ 0.74	Full spectrum (LR, GB)	Models trained on full spectra; no discrete peaks identified; highlighted need for cross-site calibration.	Weis *et al.* (2022)
CRKP versus CSKP; ColR versus ColI	2292 (830/1462) 1646 (54/1592); single center	CRKP: ACC 0.887; AUROC 0.955. Colistin: ACC 0.836; AUROC 0.845	Full spectrum (tree-based ML)	Feature importance analysis highlighted discriminative regions, including signals around *m/z* 4520–4530.	Yu *et al.* (2023)
CPK versus non-CPK; OXA-48 versus KPC typing	715 Isolates; 4547 spectra; multi-center (Spain)	CPK: ACC 97.8%; AUROC = AUPRC = 1.00. Typing: ACC 95.2%	Full spectrum (RF + SHAP)	Models trained on full spectra; SHAP analysis revealed multiple contributing signals; no universal biomarker.	Gato *et al.* (2023)
WT versus ESBL/CP	402 Isolates from two hospital domains (Spain)	AUROC ≈ 0.78 (WT); ≈ 0.90 (ESBL/CP)	Raw spectra (KSSHIBA, multi-view)	Focused on domain-shift handling and interpretability; no single-peak markers reported.	Guerrero-López *et al.* (2023)
CRKP versus CSKP	2683 (369/2314); single center	AUROC ≈ 0.91; AUPRC ≈ 0.90	Full spectrum (ANN)	Large-scale single-center benchmark; discriminatory signals identified implicitly by ANN.	Zhang *et al.* (2023)
Multi-label AMR including *K. pneumoniae*–CIP, *K. pneumoniae*–CRO	DRIAMS-A training; external validation on DRIAMS-B/C	Multi-label ≈ single-label; external generalization confirmed	Full spectrum (MLP, SVM, RF, XGBoost)	Demonstrated per-isolate multi-antibiotic prediction; no discrete peaks reported.	Astudillo *et al.* (2024)

a R and S indicate the number of resistant and susceptible isolates, respectively. ANN, artificial neural network; CRKP, carbapenemase-producing *K. pneumoniae*; ColI, colistin-intermediate; ColR, colistin-resistant; CP, carbapenemase-producing; CPK, carbapenemase-producing *K. pneumoniae*; CSKP, carbapenem-susceptible *K. pneumoniae*; ESBL, extended-spectrum β-lactamase; KSSHIBA, kernelized sparse semi-supervised interbattery Bayesian analysis; MLP, multilayer perceptron; SHAP, Shapley additive explanations; WT, wild-type.

Matrix-assisted laser desorption/ionization time-of-flight mass spectrometry (MALDI-TOF MS) has revolutionized the identification of microorganisms by providing rapid, sensitive, and accurate results (Bizzini *et al.* 2010, Stepien-Pysniak *et al.* 2017). In the MALDI-TOF MS technique, cellular peptides or proteins are ionized by a laser beam, accelerated, and separated based on their mass-to-charge ratio (*m/z*) before being analyzed by a TOF analyzer. This provides the *m/z* ratio and intensity of each sample. For the identification of bacteria, the most commonly used *m/z* range is between 2000 and 20 000, which is primarily representative of ribosomal and housekeeping proteins (Singhal *et al.* 2015). MALDI-TOF MS has great potential for identifying potential biomarkers associated with antibiotic resistance mechanisms (Chung *et al.* 2021, Wang *et al.* 2021a,b,c, Weis *et al.* 2022), although it has some disadvantages, including shifting *m/z* values and high instrument costs (Wang *et al.* 2018). Therefore, to further improve the use of MALDI-TOF MS in clinical microbiology, it is important to validate the association between the antibiotic resistance mechanism of bacteria and the mass spectral peaks (Zimmermann and Burckhardt 2017).

Researchers have increasingly explored the use of MALDI-TOF MS to identify antibiotic resistance in *K. pneumoniae*, as summarized in Table 1. Early studies focused on small cohorts and individual discriminatory peaks. Angeletti *et al.* classified 25 carbapenem-resistant isolates into two groups using ten peaks identified with ClinProTools, achieving area under the receiver operating characteristic curve (AUROC) values ranging from 0.85 to 1.0; critical discriminatory signals included those at *m/z* 9476, 4738, and 4154 (Angeletti *et al.* 2015). Flores-Treviño *et al.* analyzed 36 isolates and reported that the signal at *m/z* 6100 was absent in most carbapenem- and colistin-resistant strains, with 93.8% sensitivity but limited specificity (55.0%) (Flores-Treviño *et al.* 2019). Rocco *et al.* revealed that the signal at *m/z* 11109, corresponding to a plasmid-encoded protein, was present in most carbapenemase-producing isolates, producing 95.7% sensitivity and 100% specificity (Rocco *et al.* 2019). Building on these peak-based approaches, Huang *et al.* applied feature selection and a random forest (RF) classifier to 95 isolates, achieving 97% accuracy, with top discriminatory peaks such as *m/z* 7705, 9479, and 9541 enriched in resistant strains (Huang *et al.* 2020). However, the clinical relevance of these early studies is limited by their small sample sizes and single-center scope.

Subsequent research has moved toward larger datasets, full-spectrum machine learning (ML), and external validation. Hleba *et al.* reviewed methodological strategies, highlighting the limitations of relying on single peaks such as *m/z* 11 109 (Hleba *et al.* 2021). Wang *et al.* reported AUROC values up to 0.936 using support vector machine (SVM) models on carbapenem resistance spectra from clinical isolates (Wang *et al.* 2022). Weis *et al.* leveraged more than 300 000 spectra across four hospitals from the Database of Resistance in Antimicrobial Mass Spectra (DRIAMS) consortium, demonstrating cross-site generalization for *K. pneumoniae* resistance to CIP and CXM, although AUROCs typically remained below 0.75 (Weis *et al.* 2022). Yu *et al.* validated single-center models on 2292 isolates, reporting an AUROC of 0.955 for carbapenem resistance and extending prediction to colistin resistance (Yu *et al.* 2023). Further recent works have emphasized interpretability and generalizability: Guerrero-López *et al.* developed a Bayesian multi-view framework (KSSHIBA) to address domain shifts (Guerrero-López *et al.* 2023), while Gato *et al.* achieved near-perfect performance in classifying carbapenemase-producing isolates from 715 multi-center isolates, *albeit* without universal biomarkers (Gato *et al.* 2023). Zhang *et al.* also provided large-scale single-center evidence, who trained an artificial neural network on 2683 isolates with an AUROC of 0.91 (Zhang *et al.* 2023). Most recently, Astudillo *et al.* demonstrated a multi-label approach across DRIAMS hospitals, indicating that simultaneous modeling of multiple antibiotics, including CIP and CXM in *K. pneumoniae*, could achieve robust external generalization (Astudillo *et al.* 2024).

Despite these advancements, there remains a limited systematic investigation of resistance to CIP, CXM, and CRO in *K. pneumoniae* using MALDI-TOF MS. These antibiotics are widely used in clinical practice: CIP represents a commonly prescribed fluoroquinolone, while CXM and CRO are second- and third-generation cephalosporins frequently employed for empiric treatment of community- and hospital-acquired infections. Rising resistance rates to these agents have been associated with increased morbidity, mortality, and the need to escalate to last-resort drugs such as carbapenems and colistin. However, compared with carbapenem resistance, the early detection of resistance to CIP, CXM, and CRO has received less attention in MALDI-TOF MS research. Addressing this gap, the present study develops ML models for rapid resistance prediction against these clinically important antibiotics, incorporating feature selection to identify informative peaks and explicitly evaluating cross-site performance. By integrating interpretability with cross-hospital benchmarking, this work aims to provide clinicians with practical tools for early and accurate resistance detection, supporting more effective empiric therapy, delaying the use of last-resort agents, and contributing to antimicrobial stewardship.

## 2 Materials and methods

**Figure 1.**
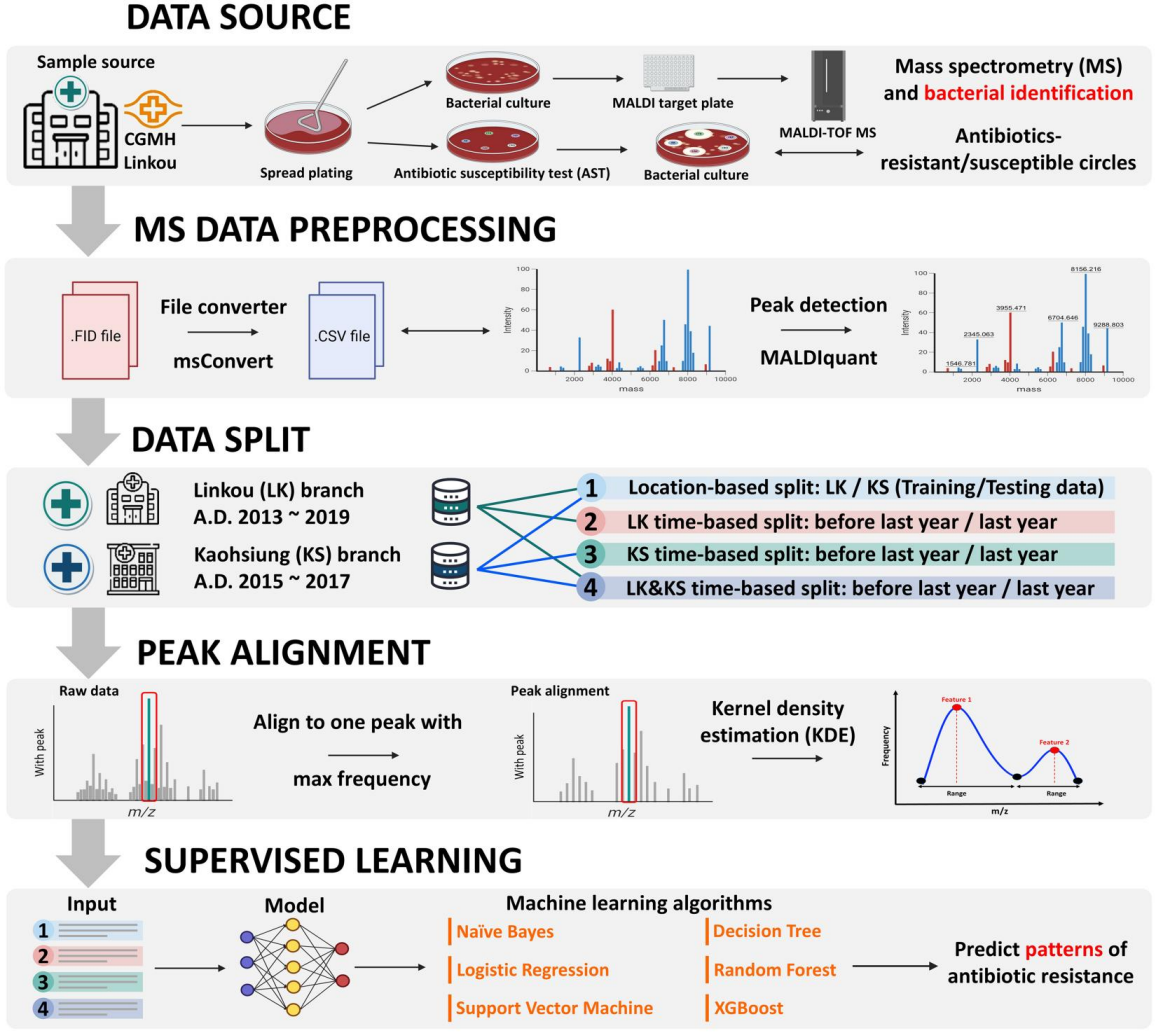
Workflow of this study. The MALDI-TOF MS of *K. pneumoniae* isolates and their corresponding AST results were obtained from Linkou Chang Gung Memorial Hospital (CGMH) and Kaohsiung CGMH. Then, we separated different datasets through location-based and time-based split and attempted to solve the shifting problem by kernel density estimation. We obtained the features of each isolate and built supervised machine learning models to predict the antibiotic resistance of the isolates.

Figure 1 illustrates the workflow employed in this study. Initially, mass spectra of *K. pneumoniae* isolates were acquired using MALDI-TOF MS, alongside their corresponding AST results. To facilitate robust validation, the datasets were stratified based on location (Linkou versus Kaohsiung) and collection year. Preprocessing included baseline subtraction, peak alignment, and kernel density estimation to mitigate potential *m/z* shift issues. Features were extracted from aligned spectra, and supervised ML models were constructed to predict resistance to CIP, CXM, and CRO.

### 2.1 Data source and dataset construction

The MALDI-TOF MS spectra of *K. pneumoniae* isolates and their corresponding AST results were obtained from Linkou (LK) Chang Gung Memorial Hospital (CGMH) and Kaohsiung (KS) CGMH. They were collected between 14 June 2013, and 29 June 2019, from the Linkou branch and between 2 February 2015, and 17 July 2017, from the Kaohsiung (KS) branch. There were three types of AST results—resistant (R), intermediate (I), and susceptible (S)—with intermediate results categorized as resistant in this study to simplify the analysis into a binary classification problem. This study was approved by the institutional review board of the Chang Gung Medical Foundation (Nos. 201901578B0 and 202101884B1). The Standards for Reporting of Diagnostic Accuracy 2015 (Bossuyt *et al.* 2015) and the Transparent Reporting of a Multivariable Prediction Model for Individual Prognosis or Diagnosis reporting guidelines (Collins *et al.* 2015) were followed.

To evaluate whether data from different sources or time points affect the accuracy of our analysis, we conducted a comprehensive investigation by developing four different datasets. The first was the location-based dataset, divided according to the two branches. The training dataset was collected from the LK branch, while the independent testing dataset was obtained from the KS branch. The second dataset was split by collection year to examine the effect of bacterial evolution over time; therefore, a time-based dataset was created for each branch. The training set and testing set for the time-based dataset in the LK branch contained data collected before 2019 and in 2019, respectively. Similarly, data collected before 2017 and in 2017 were included in the training and testing sets for the time-based dataset in the KS branch. Finally, we merged the training and independent test sets from the time-based datasets in the LK and KS branches to create a more comprehensive time-based dataset. Table 2, available as supplementary data at *Bioinformatics Advances* online, summarizes the information on the different datasets used in this study.

**Table 2.** The numbers of data and sensitive/susceptible proportion of each antibiotic and all R/S.^a^

	Linkou branch (*n = *23 367)			Kaohsiung branch (*n = *4928)		
Antibiotic	Resistant (%)	Susceptible (%)	Total	Resistant (%)	Susceptible (%)	Total
CIP	7937 (33.97%)	15 430 (66.03%)	23 367	854 (17.33%)	4074 (82.67%)	4928
CXM	10 053 (43.02%)	13 314 (56.98%)	23 367	1613 (32.73%)	3315 (67.27%)	4928
CRO	7572 (32.40%)	15 795 (67.60%)	23 367	815 (16.54%)	4074 (82.67%)	4928
All R/S	6286 (33.32%)	12 578 (66.68%)	1864	511 (13.77%)	3200 (86.23%)	3711

a All R/S, multidrug resistant or susceptible.

### 2.2 Specimen processing, bacterial species identification, and antibiotics susceptibility tests

Blood samples were collected from patients suspected of having bacteremia and cultured in trypticase soy broth (Becton Dickinson, MD, USA). Positive blood culture bottles were subcultured onto blood plate (BP) agar (Becton Dickinson, MD, USA) for AST. Individual bacterial colonies were isolated for species identification using MALDI-TOF MS (Bruker Daltonik GmbH, Bremen, Germany). Identification was confirmed for log scores >2, generated through Biotyper 3.1 software.

### 2.3 MALDI-TOF MS data analysis and peak detection

The MALDI-TOF MS measurements were performed using a Microflex LT mass spectrometer (Bruker Daltonik GmbH, Bremen, Germany) with a 60-Hz nitrogen laser. Other parameters were linear positive mode, accelerating voltage +20 kV, and 240 laser shots hit on each sample for measurement. The *K. pneumoniae* species were identified using Biotyper 3.1 (Bruker Daltonik GmbH, Bremen, Germany). We used msConvert developed by ProteoWizard (Chambers *et al.* 2012) to transform the raw MS spectra into numerical data. Finally, we implemented the “MALDIquant” package from R language to detect effective peaks mainly using the functions “removeBaseline” and “detectPeaks” for baseline subtraction and effective peak detection based on the signal-to-noise ratio, respectively (Gibb and Strimmer 2012).

### 2.4 Feature extraction

In this study, we used MALDI-TOF MS to analyze different strains of *K. pneumoniae*. Our approaches included several steps to ensure accurate and precise alignment of the mass spectra data. First, we processed the raw data of the mass spectra by rounding off the peaks of each isolate. Next, we identified the peak with the maximum frequency. In our training dataset, this peak was identified as *m/z* 3623. We used a narrow tolerance range of ±5 *m/z* around the maximum frequency peak of *m/z* 3623 to align the mass spectra of each isolate to this peak. The alignment process included only the mass spectra data that contained this peak within the tolerance range. We created a non-overlapping interval for each training dataset to simplify our data analysis. This meant that all spectra data containing only a single isolate would appear solely once in an interval. For each interval, the probability density function of the aligned mass spectrometric data was estimated by kernel density estimation (KDE) with appropriate bandwidth (Botev *et al.* 2010). We then identified the local maximum density within each interval as a feature. Finally, isolates with peaks falling within that interval were aligned to the peak with the maximum density. Based on their unique mass spectra data, we identified and differentiated the different strains of *K. pneumoniae*.

### 2.5 Model development and performance evaluation

In this study, we adopted scikit-learn (version 0.20.3) and XGBoost package (version 1.0.1) in Python (Pedregosa *et al.* 2011) to develop prediction models via various ML algorithms, including naive Bayes (NB), decision tree (DT), logistic regression (LR), SVM, RF, and extreme gradient boosting (XGB). NB is simple and computationally efficient but sensitive to irrelevant features, while DT is easy to interpret but prone to overfitting. LR is widely used but struggles with capturing complex feature relationships, and SVM handles high-dimensional data well but is sensitive to kernel function selection. Moreover, RF is robust with noisy data and captures complex relationships effectively, whilst XGBoost excels with high-dimensional data, performing well across diverse datasets. Supplementary Materials—Machine Learning Models, available as supplementary data at *Bioinformatics Advances* online, provide detailed information about these ML algorithms.

All analyses were conducted on a workstation equipped with an Intel Core i9-14900KF CPU, 128 GB RAM, and an NVIDIA RTX 2080 Ti GPU, running Windows 11 Pro. Model training typically required less than 10 minutes per antibiotic-specific dataset, and inference for a single isolate required <0.01 seconds, demonstrating that the models can be trained and executed efficiently on standard high-performance desktop hardware.

To comprehensively evaluate the ML prediction models in this study, we employed 10-fold cross-validation on the training set and independent testing on a held-out test set. A broad range of performance metrics was considered to capture both overall accuracy and robustness under class imbalance. These metrics included accuracy (ACC), sensitivity (SN), specificity (SP), the AUROC, the area under the precision-recall curve (AUPRC), the F1-score (with emphasis on the resistant class), balanced accuracy (BACC), and the Matthews correlation coefficient (MCC).

The receiver operating characteristic (ROC) curve is a two-dimensional graph that plots the true positive rate (TPR or SN) on the *y*-axis against the false positive rate (FPR = 1 − SP) on the *x*-axis at varying classification thresholds. The AUROC summarizes this trade-off, with values closer to 1.0 indicating better discrimination between resistant and susceptible isolates. The precision-recall (PR) curve plots precision (positive predictive value, PPV) against recall (SN), highlighting the ability of a model to correctly identify resistant isolates when they are relatively rare. The AUPRC is particularly useful under class imbalance, as it emphasizes performance on the positive (resistant) class rather than the majority class. For threshold-based metrics, a single cut-off was selected using Youden’s index (Youden 1950), defined as *J* = SN + SP − 1. This index identifies the threshold that maximizes the sum of sensitivity and specificity within each training fold, which was then applied to validation folds and the independent test set. The threshold-dependent metrics are defined as follows, where TP and TN are the correctly predicted resistant and susceptible isolates, and FP and FN are the incorrectly predicted resistant and susceptible isolates:


(1) $$\text{ACC}=\frac{\text{TP}+\text{TN}}{\text{TP}+\text{TN}+\text{FP}+\text{FN}}$$

(2) $$\text{SN}=\frac{\text{TP}}{\text{TP}+\text{FN}}$$

(3) $$\text{SP}=\frac{\text{TN}}{\text{FP}+\text{TN}}$$

(4) $$\text{Precision}=\frac{\text{TP}}{\text{TP}+\text{FP}}$$

(5) $$F1-\text{score}=\frac{2\cdot \;(\text{Precision}\;\cdot \;\text{SN})}{\text{Precision}\cdot \text{SN}}$$

(6) $$\text{BACC}=\frac{\text{SN}+\text{SP}}{2}$$

(7) $$\text{MCC}=\frac{\text{TP}\;\cdot \;\text{TN}-\text{FP}\;\cdot \;\text{FN}}{\sqrt{\left(\text{TP}+\text{FP})(\text{TP}+\text{FN})(\text{TN}+\text{FP})(\text{TN}+\text{FN}\right)}}$$

By reporting this comprehensive set of metrics, we ensure that model performance is not only assessed in terms of overall accuracy but also in terms of its ability to correctly detect resistant isolates, handle imbalanced datasets, and remain robust across different clinical decision thresholds. For model comparison, AUROC was used as the primary criterion, consistent with prior MALDI-TOF MS AMR studies, while AUPRC, F1-score, BACC, and MCC provide complementary perspectives on minority-class detection and balance between sensitivity and specificity.

## 3 Results

### 3.1 Summary statistics of datasets

**Figure 2.**
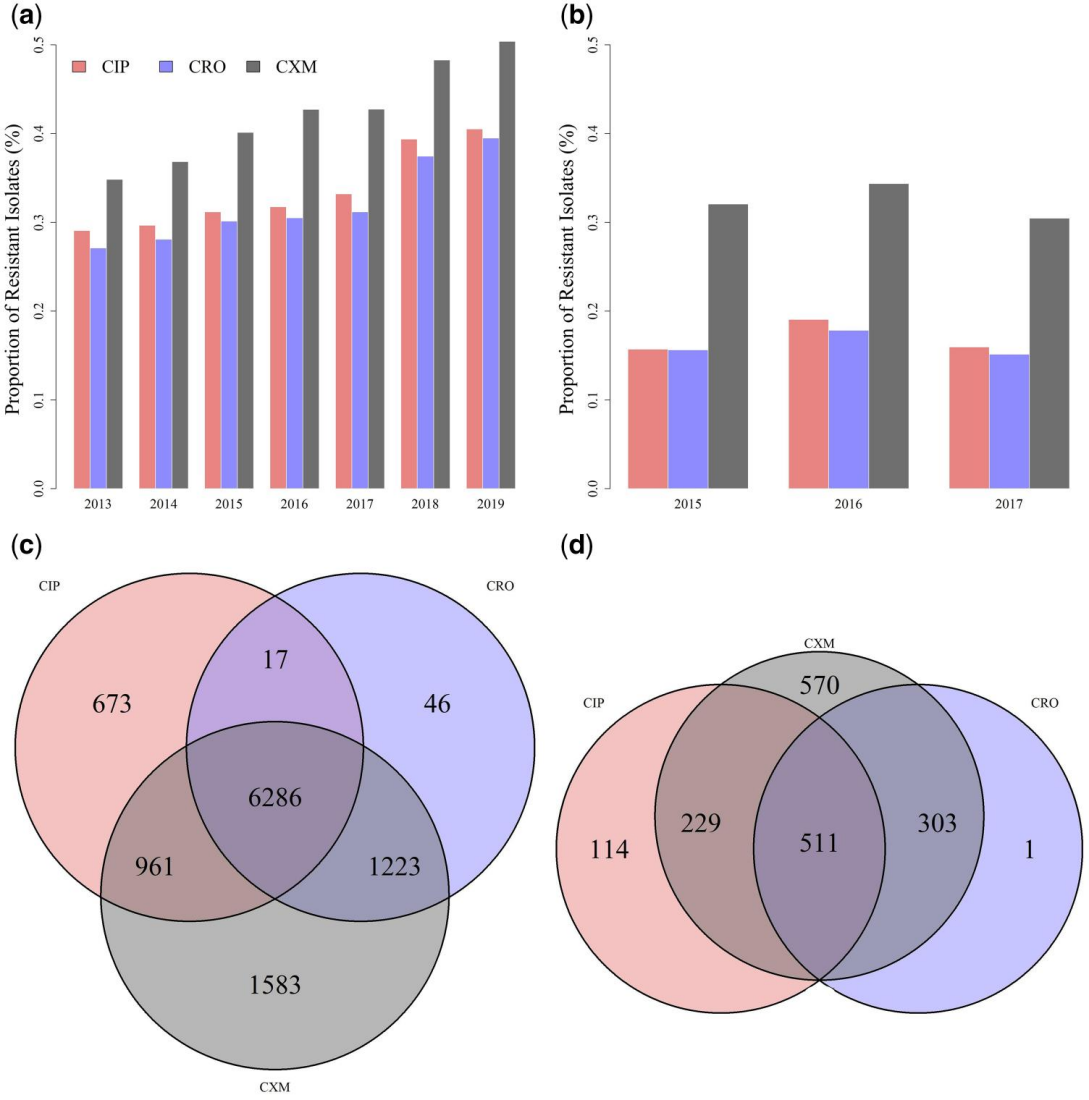
The antibiotic resistance proportions and Venn diagrams for two hospital branches. (a) Proportion of resistant isolates to ciprofloxacin (CIP), cefuroxime (CXM), and ceftriaxone (CRO) in the Linkou branch (2013–19). (b) Proportion of resistant isolates to CIP, CXM, and CRO in the Kaohsiung branch (2015–17). (c) Venn diagram showing the overlap of CIP-, CXM-, and CRO-resistant isolates in the Linkou branch. (d) Venn diagram showing the overlap of CIP-, CXM-, and CRO-resistant isolates in the Kaohsiung branch. The antibiotic labels and diagram orientations are standardized across panels (c) and (d) to allow direct comparison between branches.

In this study, *K. pneumoniae* isolates were collected from two sources: LK (*n = *23 367), and KS (*n = *4928) of CGMH. Table 2 summarizes the resistance and susceptibility proportions to three antibiotics (CIP, CXM, and CRO) and the multidrug resistance patterns of isolates that exhibited complete resistance or susceptibility to these antibiotics. The overall resistance rates for each antibiotic were higher in isolates from the LK branch than those from the KS branch. In particular, the highest proportions of CXM-resistant *K. pneumoniae* were observed in the LK and KS branches, with ratios of 43.02% and 32.73%, respectively. The proportion of multidrug-resistant isolates were notably higher in the LK branch than in the KS branch, with ratios of 33.32% and 13.77%, respectively. Figure 2a and b illustrate the two branch’s temporal trends in antibiotic resistance ratios. While the resistance ratios for each antibiotic increased over time in the LK branch, the changes were less pronounced in the KS branch. Furthermore, the resistance ratio of CXM remained higher than that of CIP and CRO in both branches. Figure 2c and d present Venn diagrams depicting the multidrug-resistant levels of *K. pneumoniae* isolates.

### 3.2 Analysis of MS spectra

The mass spectral (MS) distributions of resistant and susceptible isolates were examined, and the results are presented in Fig. 1, available as supplementary data at *Bioinformatics Advances* online. The proportion of occurrence of each *m/z* value was used to calculate the distribution of MS spectra, and values obtained from the location and time datasets were plotted in the left and right panels, respectively. To identify variants between the MS spectra of resistant and susceptible isolates, we used black lines to represent the difference between the two datasets. However, it was challenging to determine the significant differences between the MS spectra of resistant and susceptible isolates for each dataset.

**Figure 3.**
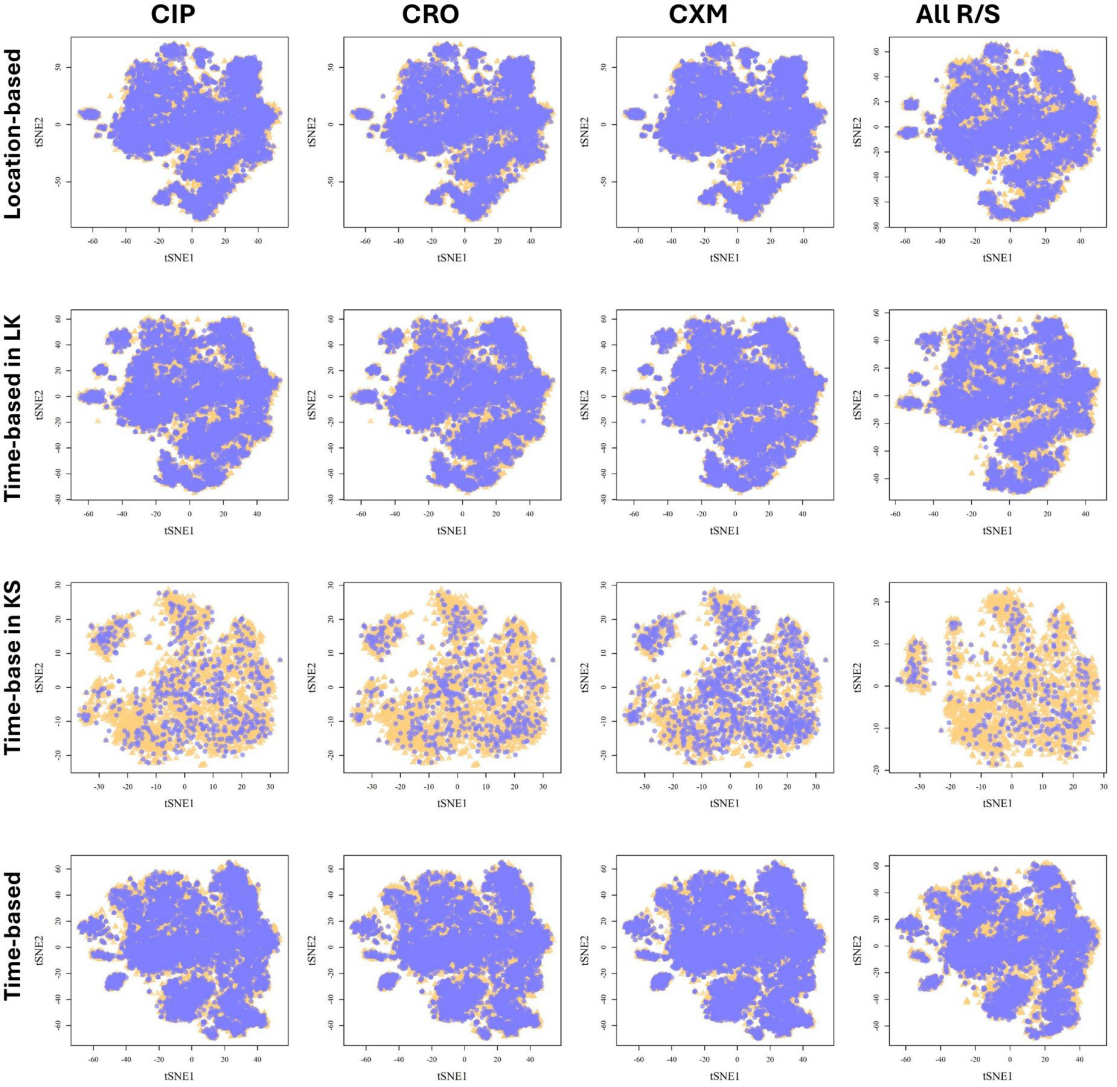
Scatter plots for t-distributed stochastic neighbor embedding with two embedded spaces on five different datasets and antibiotics. CIP, ciprofloxacin; CRO, ceftriaxone; CXM, cefuroxime.

To address this challenge, we used t-distributed stochastic neighbor embedding (t-SNE) to visualize the high-dimensional peaks of the MS spectra. Figure 3 shows the results of t-SNE with two embedded spaces, demonstrating the complexity of the data. However, the t-SNE visualization revealed further challenges in directly distinguishing between resistant and susceptible isolates. In other words, obvious areas or peaks that were resistant or sensitive were not found, even after analyzing other antibiotics. Consequently, the development of ML models is essential.

### 3.3 Performance of predictive models on different datasets

In this study, we investigated the transferability of ML models built using interhospital *K. pneumoniae* isolates via the location-based dataset. Specifically, we tested the model performance built using the LK dataset on the KS dataset. The LK dataset consisted of 23 367 *K. pneumoniae* isolates, with resistance rates of 34% for CIP, 43% for CXM, and 32% for CRO. Kernel density estimation (KDE) was used to extract 688 features from the mass spectra of each isolate. In addition, we constructed models for the multidrug-resistant or susceptible groups, denoted by all R/S, with *n = *18 864 and a resistance rate of 33%. We defined isolates resistant to all three antibiotics simultaneously as multidrug-resistant and susceptible to all three antibiotics as susceptible.

The overall AUCs for six ML models across different antibiotics and datasets are shown in Fig. 2, available as supplementary data at *Bioinformatics Advances* online. Regardless of the dataset XGBoost maintained the highest AUC among these five ML models. We therefore focused on the performance derived from XGBoost. In Table 3, available as supplementary data at *Bioinformatics Advances* online, we evaluated the accuracy of the models using 10-fold cross-validation, and the best-performing model, XGBoost, achieved an accuracy of 0.7772 ± 0.0320 for identifying all R/S. However, all the model’s independent test performance was unsatisfactory, primarily because of low sensitivity. We speculated that the models may have favored predicting susceptible isolates due to the low resistance ratio. We attempted to address this issue using a time-based split dataset at the same site. This approach allowed us to consider the temporal variability in antimicrobial resistance patterns and mitigate the shift problem caused by location differences.

**Table 3.** The performance of the time-based in both Linkou and Kaohsiung using XGBoost model (number of features = 686).

	CIP	CXM	CRO	ALL R/S
**10-Fold cross validation**				
Accuracy	0.7568 ± 0.01	0.6991 ± 0.01	0.7511 ± 0.02	0.8023 ± 0.02
Sensitivity	0.6642 ± 0.05	0.6642 ± 0.04	0.6414 ± 0.05	0.7325 ± 0.04
Specificity	0.7982 ± 0.04	0.7231 ± 0.04	0.7968 ± 0.05	0.8319 ± 0.04
AUROC	0.8074 ± 0.01	0.7681 ± 0.01	0.7919 ± 0.01	0.8622 ± 0.01
F1-score	0.6275 ± 0.63	0.6429 ± 0.64	0.6020 ± 0.60	0.6883 ± 0.69
Balanced ACC	0.7312 ± 0.73	0.6937 ± 0.69	0.7191 ± 0.72	0.7822 ± 0.78
AUPRC	0.7170 ± 0.72	0.7212 ± 0.72	0.6822 ± 0.68	0.7840 ± 0.78
MCC	0.4517 ± 0.45	0.3852 ± 0.39	0.4266 ± 0.43	0.5486 ± 0.55
**Independent test**				
Accuracy	0.7610	0.6942	0.7548	0.8001
Sensitivity	0.6876	0.7517	0.6575	0.7571
Specificity	0.7962	0.6495	0.7994	0.8207
AUROC	0.8251	0.7891	0.8101	0.8764
F1-score	0.6506	0.6827	0.6275	0.7109
Balanced ACC	0.7419	0.7006	0.7284	0.7889
AUPRC	0.7462	0.7641	0.7218	0.8211
MCC	0.4714	0.3986	0.4464	0.5614

To investigate the influence of temporal interval, we compared the performance of the models developed by time-based in LK and KS datasets. The number of *K. pneumoniae* isolates in the LK dataset was 20 896 collected this year, and 2471 in the previous year. While, the KS dataset included 4928 *K. pneumoniae* isolates, of which 3700 were collected from 2013 to 2014 and 1228 were collected in 2015. After preprocessing the mass spectra, we obtained 703 and 547 features of the peaks for the LK and KS datasets, respectively. The performance of the models developed by the time-based LK dataset is shown in Table 4, available as supplementary data at *Bioinformatics Advances* online. All training and test set models have an AUC greater than 0.7. For all R/S isolates, the best accuracy in independent tests was 0.7919. Table 5, available as supplementary data at *Bioinformatics Advances* online shows the performance of the models. However, regardless of the antibiotics used, the performance of the models was poor, with AUC values below 0.7. This could be due to low resistance ratios or limited data. Overall, the model developed from the LK dataset performed better than the KS dataset. One potential explanation could be the dataset size in LK was larger than in KS. This suggested that a model trained on a larger dataset may capture more information, leading to improved performance. In addition, we further evaluated the performance of the models constructed from an integrated dataset, which is time-based in both the LK and KS datasets.

**Table 4.** The performance of the forward feature selection in a combined dataset by time-based split using the XGBoost model.

	CIP	CXM	CRO	ALL R/S
**10-fold cross validation**				
Accuracy	0.7359 ± 0.01	0.6662 ± 0.02	0.7291 ± 0.02	0.7794 ± 0.01
Sensitivity	0.6365 ± 0.04	0.7005 ± 0.06	0.6255 ± 0.06	0.7434 ± 0.03
Specificity	0.7802 ± 0.03	0.6426 ± 0.07	0.7722 ± 0.06	0.7947 ± 0.03
AUROC	0.7818 ± 0.01	0.7411 ± 0.01	0.7690 ± 0.01	0.8491 ± 0.01
F1-score	0.5979 ± 0.60	0.6310 ± 0.63	0.5754 ± 0.58	0.6673 ± 0.67
Balanced ACC	0.7084 ± 0.71	0.6715 ± 0.67	0.6988 ± 0.70	0.7690 ± 0.77
AUPRC	0.6819 ± 0.68	0.6831 ± 0.68	0.6497 ± 0.65	0.7618 ± 0.76
MCC	0.4051 ± 0.41	0.3402 ± 0.34	0.3847 ± 0.38	0.5117 ± 0.51
**Independent test**				
Accuracy	0.7388	0.6529	0.7337	0.7902
Sensitivity	0.6174	0.7103	0.6463	0.7393
Specificity	0.7970	0.6082	0.7737	0.8147
AUROC	0.7998	0.7475	0.7929	0.8669
F1-score	0.6047	0.6417	0.6039	0.6959
Balanced ACC	0.7072	0.6592	0.7100	0.7770
AUPRC	0.7099	0.7060	0.6948	0.7974
MCC	0.4101	0.3166	0.4066	0.5388

**Table 5.** The performance of a combined dataset in the XGBoost model using an intersection set of four independent testing models (number of features = 12).

	CIP	CXM	CRO	ALL R/S
**10-Fold cross-validation**				
Accuracy	0.6928 ± 0.02	0.6618 ± 0.02	0.6770 ± 0.03	0.7209 ± 0.03
Sensitivity	0.6342 ± 0.05	0.6492 ± 0.03	0.6495 ± 0.06	0.6991 ± 0.04
Specificity	0.7190 ± 0.05	0.6704 ± 0.04	0.6884 ± 0.07	0.7302 ± 0.05
AUROC	0.7449 ± 0.01	0.7220 ± 0.01	0.7350 ± 0.01	0.7889 ± 0.01
F1-score	0.5600 ± 0.56	0.6106 ± 0.61	0.5419 ± 0.54	0.5992 ± 0.60
Balanced ACC	0.6766 ± 0.68	0.6598 ± 0.66	0.6690 ± 0.67	0.7146 ± 0.71
AUPRC	0.6244 ± 0.62	0.6638 ± 0.66	0.5906 ± 0.59	0.6715 ± 0.67
MCC	0.3362 ± 0.34	0.3162 ± 0.32	0.3165 ± 0.32	0.4037 ± 0.40
**Independent test**				
Accuracy	0.7332	0.6683	0.7178	0.7521
Sensitivity	0.5013	0.6362	0.5164	0.6084
Specificity	0.8441	0.6933	0.8100	0.8212
AUROC	0.7527	0.7352	0.7355	0.7984
F1-score	0.5487	0.6267	0.5348	0.6145
Balanced ACC	0.6727	0.6647	0.6632	0.7148
AUPRC	0.6412	0.6990	0.6061	0.7059
MCC	0.3650	0.3285	0.3330	0.4319

There were 24 596 data points in the training set and 3699 data points for each antibiotic in the testing set. We also had 19 634 data points in training and 2941 in testing for all R/S groups. After preprocessing the mass spectra, we extracted 686 features and used them to develop models. Table 3 shows the performance of the models. The highest independent test AUC and accuracy achieved were 0.8764 and 0.8001, respectively, for the XGBoost model utilizing all R/S isolates and 686 features.

### 3.4 Investigations of the selected features

**Figure 4.**
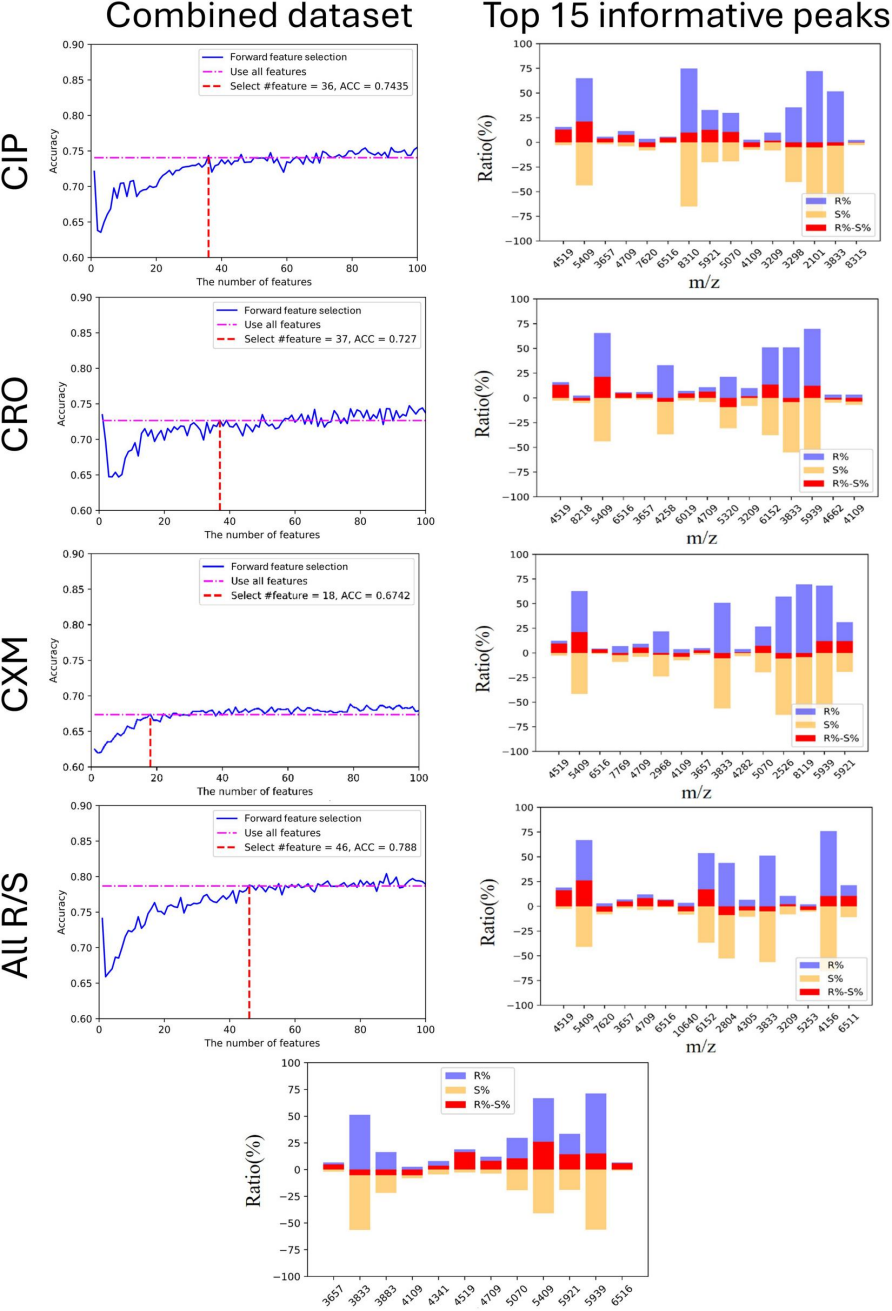
The upper left panel shows the 10-fold cross-validation results of forward feature selection in a combined dataset by time-based split. The upper right panel shows the resistant and susceptible rates in the top 15 informative peaks listed in feature importance order. The bottom figure shows the resistant and susceptible rates in all R/S of 12 informative peaks from an intersection set of four models in Table 6; listed in ascending order of *m/z*. CIP, ciprofloxacin; CRO, ceftriaxone; CXM, cefuroxime; R, resistant; S, susceptible.

The features were too copious to evaluate the importance of informative peaks. Additionally, a large number of features in a model can increase the risk of overfitting and significantly extend the time required to fit the data. Thus, we implemented feature selection to reduce the dimensionality of the features. Initially, we ranked the features from the independent testing models via time-based for both the LK and KS datasets across each antibiotic and all R/S groups. Subsequently, a forward feature selection strategy was applied. Features were selected such that the model’s accuracy was equal to or greater than that of models utilizing all features while minimizing the number of features to >1. The feature selection process is illustrated in the upper left panel of Fig. 4, and the corresponding evaluation metrics for training and independent testing at the selected point are summarized in Table 4. The number of selected features in each independent testing model was 36, 18, 37, and 46 for CIP, CXM, CRO, and all R/S groups, respectively. While the number of features was reduced and approximately retained the 10-fold cross-validation performance, a slight decrease in independent testing performance was observed. In addition, the top 15 informative peaks based on feature importance for each independent testing model are displayed in the upper right panel of Fig. 4, with peak at *m/z* 4519 identified as the most significant across all models.

To investigate features related to antibiotic resistance, we applied additional feature sets to the combined dataset. The intersection of features from the four independent testing models for CIP, CRO, CXM, and all R/S datasets yielded 12 features. The performance metrics and corresponding features are listed in Tables 5 and 6. In the independent testing performance for all R/S datasets, the highest AUC and accuracy were 0.8000 and 0.7525, slightly lower than the performance achieved with 46 features, where the AUC and accuracy were 0.8622 and 0.7858, respectively.

**Table 6.** The performance of the forward feature selection in a combined dataset by time-based split using the XGBoost model.

Peak (*m/z*)	CIP		CXM		CRO		All R/S	
	R%	S%	R%	S%	R%	S%	R%	S%
3657.27	5.73	1.85	4.66	1.94	5.85	1.88	6.81	1.91
3832.91	51.65	55.02	50.73	56.22	51.02	55.21	51.23	56.40
3883.47	16.74	21.39	17.74	21.49	17.18	21.11	16.50	21.74
4109.42	2.74	7.49	3.70	7.63	3.38	7.13	2.57	7.95
4341.15	7.86	4.38	6.70	4.60	7.42	4.64	8.10	4.52
4518.92	15.47	2.64	12.27	2.69	15.90	2.73	18.93	2.62
4709.23	11.29	3.90	9.33	4.01	10.66	4.32	12.07	3.81
5069.92	29.85	19.26	26.78	19.59	27.60	20.42	29.78	19.23
5408.99	64.99	43.85	62.89	41.73	65.51	44.08	66.93	40.93
5920.54	32.75	20.27	31.15	19.27	31.89	20.89	33.40	18.98
5938.82	69.00	57.72	68.23	56.35	69.88	57.59	71.29	56.15
6516.21	5.53	0.88	4.41	0.87	5.67	0.92	6.83	0.87

For the peaks selected from the intersection set of four models, we observed that the percentage of resistant isolates (R%) for nine peaks—*m/z* 3657, 4341, 4519, 4709, 5070, 5409, 5921, 5939, and 6516—was higher than the percentage in susceptible isolates (S%), except for three peaks: *m/z* 3833, 3883, and 4109. Further analysis of these informative peaks revealed the resistant and susceptible percentages (R% and S%) for the 12 peaks (Fig. 4). The figure also highlights the difference between R% and S% (R% – S%) within a range of ±5. Peaks with a much larger difference and a low S% were found to have a stronger and more significant association with antibiotic resistance, providing valuable insights into the resistance mechanisms.

To explore alternative feature sets, we incorporated peaks identified in several previous studies (Angeletti *et al.* 2015, Flores-Treviño *et al.* 2019, Rocco *et al.* 2019, Huang *et al.* 2020), resulting in a total of 22 features. The raw mass spectra of all isolates were aligned to a peak at *m/z* 3623 ± 5, and features were defined based on the interval within the ±5 *m/z* range around the corresponding peak. However, as shown in Table 7, available as supplementary data at *Bioinformatics Advances* online, the independent test performance of these features yielded AUC values below 0.7, regardless of the antibiotic used. It was observed that the peaks identified in previous studies were primarily associated with carbapenem or colistin resistance, which differs from the focus of our study. Nevertheless, the independent testing for all R/S isolates achieved a sensitivity of 0.6901 and a specificity of 0.5624. As shown in Table 8, available as supplementary data at *Bioinformatics Advances* online, the percentage of resistant and susceptible isolates for most of these peaks exceeded 30% and 90%, respectively. However, there was no significant difference in the percentages between resistant and susceptible isolates for most peaks, except for a few. Notably, the peak at *m/z* 11109 exhibited lower percentages for both resistant and susceptible isolates compared to other peaks, likely due to the strict peak detection criteria applied using the MALDIquant R package.

We also investigated five informative peaks proposed by Bar-Meir *et al.* related to ESBL-*K. pneumoniae* (Bar-Meir *et al.* 2020). As shown in Fig. 3, available as supplementary data at *Bioinformatics Advances* online, no significant differences were observed between the resistant and susceptible percentages across all R/S isolates, suggesting that these peaks were not critical for distinguishing multiple antibiotic resistance in *K. pneumoniae* isolates. In contrast, as shown in Table 5, we identified 12 peaks associated with antibiotic resistance that demonstrated superior scoring criteria compared to the 22 peaks proposed in the previous study. Furthermore, the peaks proposed in our study exhibited greater potential to differentiate multiple antibiotic resistance in *K. pneumoniae* isolates, as evidenced by a clearer distinction in the percentage of resistant and susceptible isolates compared to the peaks proposed in earlier research.

## 4 Discussion and conclusion

In this study, we demonstrate that using ML models and MALDI-TOF MS spectra can provide rapid, interpretable antibiotic resistance predictions in *K. pneumoniae* for CIP, CXM, and CRO. Using large, multi-year datasets from two hospitals, the best models achieved independent test accuracy of 0.7858 (sensitivity 0.7289; specificity 0.8127), with complementary imbalance-aware metrics (AUROC/AUPRC, F1, balanced accuracy, and Matthews correlation coefficient) reported alongside thresholded operating points. These results indicate that MALDI-TOF–based models can contribute to earlier resistance risk stratification for these commonly used agents.

Generalizability analyses revealed a consistent pattern: performance remained relatively stable in time-based evaluations within a site, but decreased under cross-hospital transfer. This behavior is compatible with domain shift arising from biological and technical sources. Biologically, hospitals can differ in clonal structure, resistance-gene repertoires (for example, ESBL families), and local selection pressures, which together influence phenotype distributions. Technically, site-specific workflows (specimen handling, media, instrument settings) can alter spectral characteristics despite baseline subtraction, normalization, and anchor-based alignment. These observations underscore the importance of site-aware evaluation and suggest that local calibration or modest fine-tuning with representative isolates is prudent for deployment.

With respect to model interpretability, we report a set of resistant-enriched spectral signals, for example, *m/z* 3657, 4341, 4519, 4709, 5070, 5409, 5921, 5939, 6516, that were more frequent among resistant isolates across analyses. We emphasize that these *m/z* values are phenotypic correlates that help explain model behavior; they are not presented as causal mechanisms in the MALDI-TOF linear mode used here. To quantify their support, we combined model-based importance with univariate testing under multiple-comparison control and examined consistency across splits, including intersection-based feature sets. Definitive attribution of peaks to peptides or proteins will require orthogonal validation, for example, targeted MS/MS or integration with genomic data, which we identify as an important next step.

Several factors probably explain the differences between our results and some higher headline metrics reported elsewhere. First, many prior studies address different endpoints, for example, carbapenemase production, or rely on single-center internal validation; our emphasis on drug-specific resistance (CIP/CXM/CRO) and external, cross-site testing provides a stricter—and more clinically realistic—assessment of transportability. Second, we adopted conservative, leakage-resistant procedures (all preprocessing and thresholding within folds; independent held-out tests) and reported imbalance-aware metrics, choices that tend to yield more tempered but robust estimates. Finally, our feature selection prioritized stability and interpretability across datasets, which may trade marginal discrimination for reproducibility.

This work has some limitations. MALDI-TOF MS spectra were acquired in linear positive mode without fragmentation, precluding confident peptide/protein identification. Class imbalance varied by antibiotic and site, which we mitigated through stratified sampling, class-weighted training where available, and reporting of imbalance-aware metrics. However, this can still affect sensitivity in external tests. Although we examined location- and time-based splits, prospective multi-site validation and assessment under additional instruments and workflows are warranted.

In summary, ML models trained on MALDI-TOF MS spectra could support rapid resistance risk stratification for CIP, CXM, and CRO in *K. pneumoniae*, particularly when calibrated with local data. Future work will focus on (i) prospective multi-site evaluation and lightweight adaptation strategies for cross-hospital deployment, (ii) orthogonal validation of resistant-enriched *m/z* signals using targeted proteomics and/or genomic integration, and (iii) exploration of domain-adaptation methods to improve transportability further. These directions aim to translate spectral pattern recognition into reliable, locally adaptable decision support for antimicrobial stewardship.

## Supplementary Material

vbaf303_Supplementary_Data

## Data Availability

The data underlying this article are available on request from the authors.
