# Supplementary material for: AI-powered rapid detection of multidrug-resistant Klebsiella pneumoniae with informative peaks of MALDI-TOF MS
Source: Bioinform Adv. 2025 Nov 24;6(1):vbaf303. doi: 10.1093/bioadv/vbaf303 (PMC12776345; doi:10.1093/bioadv/vbaf303)
Supplement: vbaf303_Supplementary_Data [file vbaf303_supplementary_data.pdf]

AI-powered Rapid Detection of Multidrug-Resistant *Klebsiella pneumoniae* with Informative Peaks of MALDI-TOF MS

Jang-Jih Lu[1,2], Hsin-Yao Wang[2], Chia-Ru Chung[3], Yun Tang[4], Ming-Chien Chiang[3], Li-Ching Wu[5], Justin Bo-Kai Hsu[6], Tzong-Yi Lee[4,7], and Jorng-Tzong Horng[3,*]

[1]Department of Laboratory Medicine, Taipei Tzu Chi Hospital, Buddhist Tzu Chi Medical Foundation, New Taipei City 23142, Taiwan

[2]Department of Laboratory Medicine, Chang Gung Memorial Hospital at Linkou, Taoyuan, Taiwan

[3]Department of Computer Science and Information Engineering, National Central University, Taoyuan, Taiwan

[4]Institute of Bioinformatics and Systems Biology, National Yang Ming Chiao Tung University, Hsinchu, Taiwan

[5]Department of Biomedical Sciences and Engineering, National Central University, Taoyuan, Taiwan

[6]Department of Computer Science and Engineering, Yuan Ze University, Taoyuan, Taiwan

[7]Center for Intelligent Drug Systems and Smart Biodevices (IDS^{2}B), National Yang Ming Chiao Tung University, Hsinchu City, Taiwan

*Corresponding author. Department of Computer Science and Information Engineering, National Central University, No. 300, Zhongda Rd., Zhongli District, Taoyuan City, Taiwan. E-mail: horng@db.csie.ncu.edu.tw

Supplementary Materials

Machine Learning Models

Naïve Bayes (NB)

Naïve Bayes was based on Bayes' theorem with naïve assumption of conditional independence between every pair of features [22]. Given class variable y and dependent feature x_1 through x_n, the following relationship states the Bayes' theorem:

$$P(y \mid x_1, \dots, x_n) = \frac{P(y)P(x_1,\dots,x_n \mid y)}{P\ (x_1,\dots,x_n)} \quad (1)$$

Using the naïve assumption of conditional independence, then we can get:

$$P(x_i \mid y, x_1, \dots, x_{i-1}, x_{i+1}, \dots, x_n) = P(x_i \mid y)\ \forall i = 1, \dots, n \quad (2)$$

Then we used Gaussian Naive Bayes algorithm in this study with the likelihood of the features assumed to be Gaussian with *'GaussianNB'* function in scikit-learn '*naive_bayes'* package as follow:

$$P(x_i \mid y) = \frac{1}{\sqrt{2\pi\sigma_y{}^2}}\, exp\left(-\frac{(x_i-\mu_y)^2}{2\sigma_y{}^2}\right) \quad \forall i = 1, \dots, n \quad (3)$$

The parameters σ_y and μ_y are estimated using maximum likelihood. Since $P\ (x_1, \dots, x_n)$ is constant given the input, then we can use Maximum A Posteriori (MAP) estimation to estimate $P(y)$ and $P(x_i \mid y)$. Finally, we can get the classification results to the highest $P(x_i \mid y)$.

Logistic Regression (LR)

Logistic Regression (LR) is a traditional and classic statistical model assumed that the input variables are continuous measurement, especially appropriate for binary classification. The prediction of LR is to use logistic function through a linear combination of multiple variables. Supposed the class variables $Y = \{1,0\}$ and features X, the following is the formula of LR:

$$logit[p(X)] = log\left(\frac{p(X)}{1-p(X)}\right) = \alpha + \beta X \quad (4)$$

Then we can get:

$$p(X) = \left(\frac{e^{\alpha+\beta X}}{1-e^{\alpha+\beta X}}\right) \quad (5)$$

In simplest case, if the decision boundary is 0.5, then the prediction result is observed as the 'positive' class $Y = 1$ when

$$p(X) \geq 0.5 \tag{6}$$

and the 'negative' class $Y = 0$ otherwise. Besides, we implemented this algorithm by *'LogisticRegression'* function to predict probability using the parameter 'solver=warn' in scikit-learn *'linear_model'* package.

Decision Tree (DT)

Decision Tree (DT) is a tree-based classifier whose leaves represent class labels and branches represent condition of features that lead to class labels. There are three commonly used impurity measures used in binary decision trees: Entropy, Gini index, and Classification Error. In this study, the function *'DecisionTreeClassifier'* with parameter *'criterion=Gini'* was used in scikit-learn 'tree' package. The following is the formula of Gini index:

$$Gini = 1 - \sum_{j}^{m} {p_j}^2 \tag{7}$$

where p_j is the probability of class j. The Gini index is maximal if the classes are trained well in the model. Then we can use decision algorithm to start from tree root and split the data based on features, resulting in the largest information gain (IG). The following is a definition of IG:

$$IG(D_p) = I(D_p) - \frac{N_{left}}{N_p} I(D_{left}) - \frac{N_{right}}{N_p} I(D_{right}) \tag{8}$$

where I could be Gini index, D_p , D_{left}, and D_{right} are the dataset of the parent, left and right child node.

Support Vector Machine (SVM)

SVM is a supervised machine learning classifier. This method is to estimate the hyperplane for separating different classes and maximizing their margins between them. For example, given training point sets:

$$\{(x_i, y_i)\}\ \forall i = 1, \ldots, n\ , where\ x_i\ \in\ R^d\ , y_i\ \in\ \{\pm 1\}. \tag{9}$$

We hope to find a line $f(x) = w^T x - b$ to separate two classes such that:

$$\{(x_i, y_i)\}\ \in \left\{\{(x_j, y_j)\} \mid f(x_j) \geq 1,\ 0 \leq j \leq n\right\}\ or$$
$$\left\{\{(x_k, y_k)\} \mid f(x_k) \leq -1\ , 0 \leq k \leq n\right\} \tag{10}$$

where w is the boundary hyperplane normal vector. And we hope to let the margins between two support vectors as max as possible, and we can transform this optimization

problem to the following formula:

$$\max_{w}\left\{\frac{2}{||w||}\right\} \rightarrow \min_{w}\frac{1}{2}w^T w$$

$$subject\ to\ \ y_i(w^T x_i - b) \geq 1, \forall i = 1, \dots, n \qquad (11)$$

Therefore, we get the hyperplane $f(x)$ for the sets $\{(x_i, y_i)\}$. In addition, we used *'SVC'* function with *'kernel=rbf'* to predict output probability using the parameter *'probability = True'* in scikit-learn *'svm'* package.

Random Forest (RF)

Intuitively, RF is an ensemble learning method composed of many decision trees. We implemented in the function *'RandomForestClassifier'* with the parameters *'n_estimators = 200'* of scikit-learn 'ensemble' package. Generally speaking, the more the number of trees (corresponding to the parameters *'n_estimators'*), the better the performance of the models, but with long operating time. We used Gini index as the criterion of impurity in each partition in this study. Other parameters setting such as *'min_sample_split = 2'* which is the minimum number of splits in the internal nodes, *'min_samples_leaf = 1'* which is the minimum number of leaf splitting.

Extreme Gradient Boosting (XGBoost)

XGBoost is an efficient supervised machine learning method developed by Chen et. al. [23]. This method is based on a scalable tree boosting system combining the concepts of cache access patterns, data compression and sharding for using minimal resources. It also supports parallel tree learning to reduce training time. The training process of XGBoost is to optimize the objective function composed of training loss and regularization. Using additive functions is the main strategy of XGBoost. For example, given the dataset with n samples and m features, $D = \{(x_i, y_i)\}$ $(|D| = n, x_i \in R^m, y_i \in R)$, a predictive value $\mathrm{y_i}$ using K additive functions defined as follow:

$$\widehat{y_i} = \sum_{k=1}^{K} f_k(x_i), f_k \in F \qquad (12)$$

where $F = \{f(x) = \omega_{q(x)}\}$ $(q: R^m \rightarrow T, m \in R^T)$ is the space of regression trees, also called CART. Here q is the structure of each tree with the number of trees T. A function f_k corresponds to a tree structure q and leaf weight w. The ω_i stands for the score on $i\text{-}th$ leaf. To learn the functions used in the model, we need the following regularized objective function and minimize it:

$$obj = \sum_i l(y_i, \hat{y_i}) + \sum_k \Omega(f_k) \;\; where \; \Omega(f) = \gamma T + \frac{1}{2}\lambda \|\omega\|^2 \qquad (13)$$

Here l is a loss function that measures the difference between the prediction $\hat{y_i}$ and the target y_i. The second term Ω penalizes the complexity of the model where T is the number of leaves. γ and λ are tunable parameters. We implemented in the function *'xgb.XGBClassifier'* with the parameters *'n_estimators = 100'* and *' objective="binary:logistic"'* of *'xgboost'* package.

Supplementary Figures

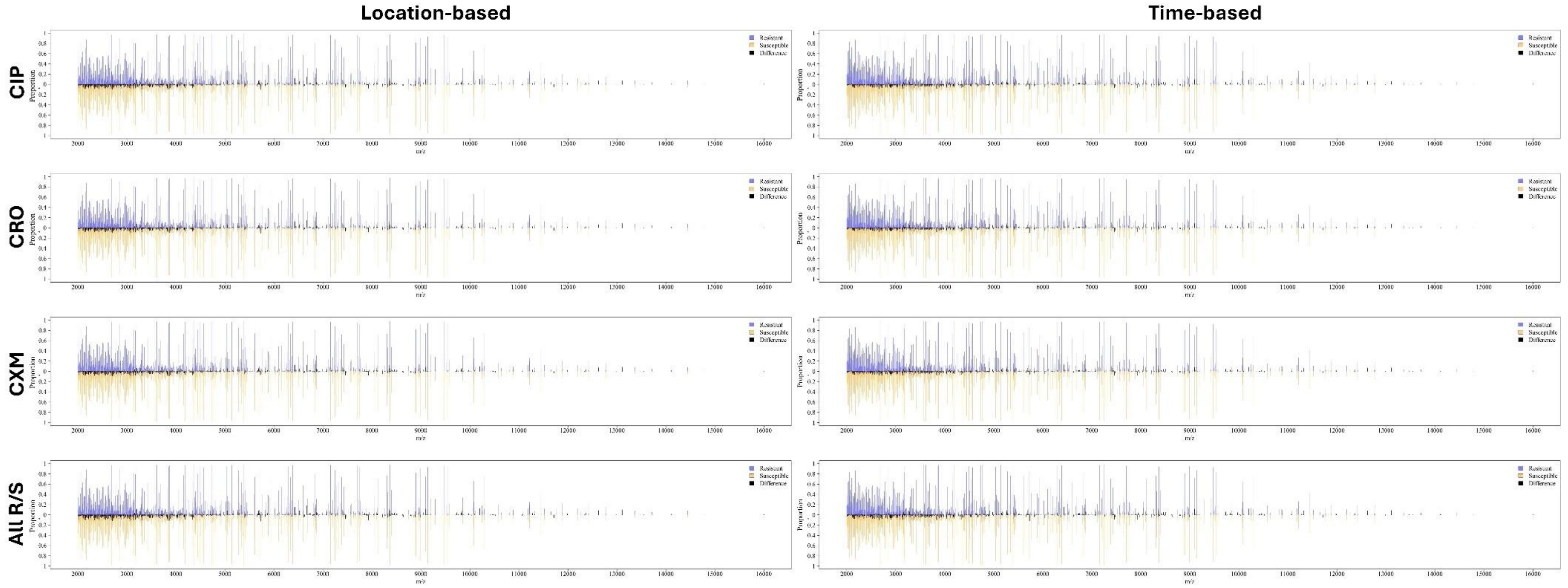

Supplementary Figure S1. Comparison of spectra of the resistant and susceptible strains for the different datasets and antibiotics. Purple: resistance, yellow: susceptible, black: difference.

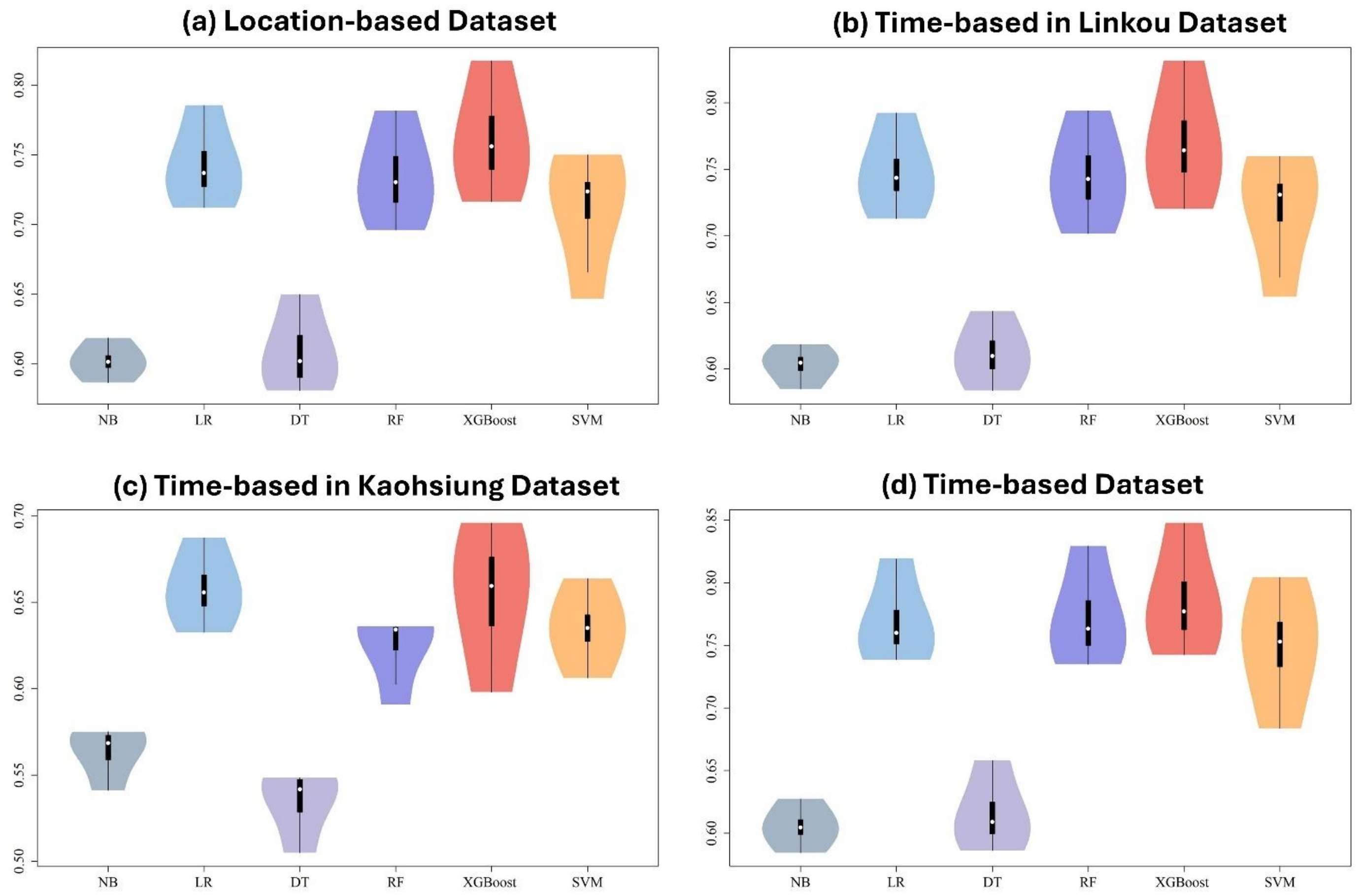

Supplementary Figure S2. The violin plots for the average area under the receiver operating characteristic curves derived from six machine learning models among different antibiotics and datasets. NB: naïve Bayes; DT: Decision tree; LR: Logistic regression; SVM: Support vector machine; RF: random forest.

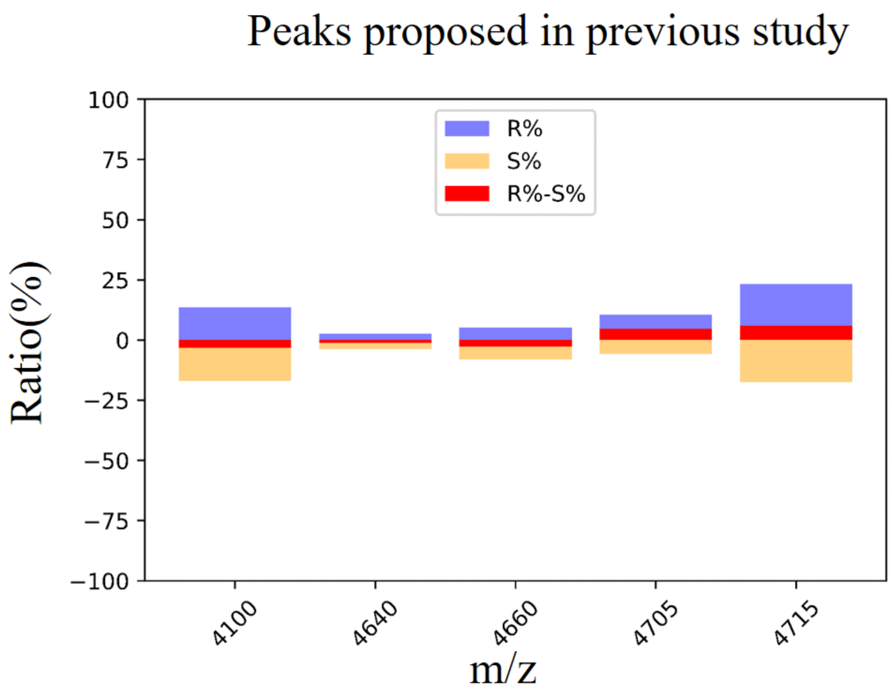

Supplementary Figure S3. The resistant and susceptible ratio in all R/S of 5 informative peaks proposed by Bar-Meir et al. [17] and listed in ascending order of m/z

Supplementary Tables

Supplementary Table S1. The characteristics of three antibiotics often used in *K. pneumoniae infection*.

Antibiotic	Class	Generation	Mechanism of action
Ciprofloxacin (CIP)	fluoroquinolone	second	DNA gyrase inhibitor
Cefuroxime (CXM)	cephalosporin	second	bacterial cell wall synthesis inhibitor
Ceftriaxone (CRO)		third	

Supplementary Table S2. Information of the datasets used in this study.

Name of dataset	Source of training data	Source of independent testing data
Location-based	From the Linkou branch at any time	From the Kaohsiung branch at any time
Time-based in Linkou	From the Linkou branch before 2019	From the Linkou branch in 2019
Time-based in Kaohsiung	From the Kaohsiung branch before 2017	From the Kaohsiung branch in 2017
Time-based in both Linkou and Kaohsiung	From the Linkou branch before 2019 and the Kaohsiung branch before 2017	From the Linkou branch in 2019 and the Kaohsiung branch in 2017

Supplementary Table S3. The numbers of data and sensitive/susceptible proportion of each antibiotic and all R/S.

	CIP	CXM	CRO	ALL R/S
	**10-fold cross validation**			
**Accuracy**	0.7629±0.01	0.7110±0.01	0.7524±0.02	0.8047±0.01
**Sensitivity**	0.6596±0.02	0.6709±0.06	0.6735±0.05	0.7469±0.03
**Specificity**	0.8161±0.02	0.7413±0.06	0.7902±0.05	0.8335±0.02
**AUROC**	0.8162±0.01	0.7831±0.01	0.8063±0.01	0.8728±0.01
**F1-score**	0.6540±0.65	0.6654±0.67	0.6378±0.64	0.7182±0.72
**Balanced ACC**	0.7378±0.74	0.7061±0.71	0.7319±0.73	0.7902±0.79
**AUPRC**	0.7481±0.75	0.7544±0.75	0.7213±0.72	0.8188±0.82
**MCC**	0.4743±0.47	0.4150±0.41	0.4547±0.45	0.5709±0.57
	**Independent test**			
**Accuracy**	0.7948	0.6769	0.8005	0.8359
**Sensitivity**	0.3548	0.3639	0.2147	0.3425
**Specificity**	0.8871	0.8293	0.9166	0.9147
**AUROC**	0.7060	0.6874	0.6618	0.7514
**F1-score**	0.3748	0.4244	0.2626	0.3650
**Balanced ACC**	0.6209	0.5966	0.5657	0.6286
**AUPRC**	0.3722	0.5068	0.288	0.3832
**MCC**	0.2531	0.2141	0.1591	0.2720

Supplementary Table S4. The performance of time-based in Linkou dataset (number of features = 703).

Antibiotic	#Data (R%)	Accuracy	Sensitivity	Specificity	AUC
10-fold cross validation (combined dataset before last year)					
CIP	20,896 (33%)	0.7390±0.0277	0.6077±0.0814	0.8042±0.0531	0.7715±0.0375
CXM	20,896 (42%)	0.6758±0.0260	0.5698±0.1370	0.7531±0.0874	0.7204±0.0520
CRO	20,896 (32%)	0.7278±0.0350	0.5953±0.1018	0.7889±0.0760	0.7571±0.0435
All R/S	16,680 (32%)	0.7742±0.0249	0.7005±0.0612	0.8094±0.0357	0.8317±0.0338
Independent test (combined dataset in last year)					
CIP	2,471 (41%)	0.7596	0.6444	0.8381	0.8278
CXM	2,471 (50%)	0.6981	0.6048	0.7928	0.7833
CRO	2,471 (40%)	0.7552	0.6291	0.8375	0.8180
All R/S	2,004 (42%)	0.7919	0.7286	0.8376	0.8780

Note. CIP: Ciprofloxacin; CXM: Cefuroxime; CRO: Ceftriaxone; AUC: Area under the receiver operating characteristic curve.

Supplementary Table S5. The performance of time-based in Linkou dataset (number of features = 703).

Antibiotic	#Data (R%)	Accuracy	Sensitivity	Specificity	AUC
10-fold cross validation (combined dataset before last year)					
CIP	20,896 (33%)	0.7390±0.0277	0.6077±0.0814	0.8042±0.0531	0.7715±0.0375
CXM	20,896 (42%)	0.6758±0.0260	0.5698±0.1370	0.7531±0.0874	0.7204±0.0520
CRO	20,896 (32%)	0.7278±0.0350	0.5953±0.1018	0.7889±0.0760	0.7571±0.0435
All R/S	16,680 (32%)	0.7742±0.0249	0.7005±0.0612	0.8094±0.0357	0.8317±0.0338
Independent test (combined dataset in last year)					
CIP	2,471 (41%)	0.7596	0.6444	0.8381	0.8278
CXM	2,471 (50%)	0.6981	0.6048	0.7928	0.7833
CRO	2,471 (40%)	0.7552	0.6291	0.8375	0.8180
All R/S	2,004 (42%)	0.7919	0.7286	0.8376	0.8780

Note. CIP: Ciprofloxacin; CXM: Cefuroxime; CRO: Ceftriaxone; AUC: Area under the receiver operating characteristic curve.

Supplementary Table S6. The performance of time-based in Kaohsiung dataset (number of features = 547).

Antibiotic	#Data (R%)	Model	Accuracy	Sensitivity	Specificity	AUC
10-fold cross validation (combined dataset before last year)						
CIP	3,700 (18%)	XGBoost	0.6449±0.0895	0.6416±0.1490	0.6457±0.1379	0.6695±0.0417
CXM	3,700 (33%)	LR	0.6127±0.0605	0.6942±0.1073	0.5717±0.1323	0.6530±0.0439
CRO	3,700 (17%)	LR	0.6359±0.0848	0.6139±0.1156	0.6405±0.1240	0.6326±0.0249
All R/S	2,774 (14%)	XGBoost	0.6868±0.0962	0.6517±0.1288	0.6926±0.1331	0.6960±0.0389
Independent test (combined dataset in last year)						
CIP	1,228 (16%)	XGBoost	0.6474	0.5816	0.6599	0.6683
CXM	1,228 (30%)	LR	0.5521	0.8021	0.4426	0.6802
CRO	1,228 (15%)	LR	0.5440	0.6989	0.5163	0.6514
All R/S	937 (12%)	XGBoost	0.7193	0.5043	0.7494	0.6688

Note. CIP: Ciprofloxacin; CXM: Cefuroxime; CRO: Ceftriaxone; AUC: Area under the receiver operating characteristic curve.

Supplementary Table S7. The performance of the time-based in both Linkou and Kaohsiung using XGBoost model using peaks proposed by Angeletti et al., Flores-Trevino et al., Rocco et al., and Huang et al. [12-15] (number of features = 22).

Antibiotic	#Data (R%)	Accuracy	Sensitivity	Specificity	AUC
10-fold cross validation (combined dataset before last year)					
CIP	24,596 (31%)	0.6096±0.0327	0.5526±0.1039	0.6351±0.0883	0.6220±0.0277
CXM	24,596 (41%)	0.5936±0.0218	0.5717±0.0727	0.6087±0.0774	0.6197±0.0240
CRO	24,596 (29%)	0.6205±0.0423	0.5085±0.1237	0.6671±0.1093	0.6125±0.0227
All R/S	19,634 (30%)	0.6243±0.0501	0.6243±0.0804	0.6243±0.1011	0.6634±0.0278
Independent test (combined dataset in last year)					
CIP	3,699 (32%)	0.5977	0.6082	0.5927	0.6377
CXM	3,699 (44%)	0.5858	0.6368	0.5462	0.6336
CRO	3,699 (31%)	0.6083	0.5611	0.6299	0.6288
All R/S	2,941 (32%)	0.6039	0.6901	0.5624	0.6808

Note. CIP: Ciprofloxacin; CXM: Cefuroxime; CRO: Ceftriaxone; AUC: Area under the receiver operating characteristic curve.

Supplementary Table S8. The ratio with peaks proposed by Angeletti et al., Flores-Trevino et al., Rocco et al., and Huang et al. [12-15].

	CIP		CXM		CRO		All R/S	
m/z	R%	S%	R%	S%	R%	S%	R%	S%
2636.88	74.11	73.80	73.96	73.85	74.21	73.77	74.46	73.88
4154.00	75.81	67.49	73.85	67.43	75.16	67.94	77.15	67.23
4362.22	94.13	94.62	94.08	94.74	94.17	94.59	94.06	94.77
4738.00	96.88	97.07	96.93	97.06	96.82	97.09	96.73	97.09
4768.28	95.94	94.87	95.36	95.09	95.54	95.06	95.74	95.03
4770.00	97.97	97.47	97.72	97.55	97.84	97.53	97.95	97.56
5379.42	95.42	94.79	95.01	94.96	95.46	94.78	95.50	94.95
5381.00	98.31	97.89	98.09	97.98	98.23	97.94	98.29	97.98
6096.00	44.64	46.24	44.90	46.33	44.39	46.31	43.85	46.22
6100.00	51.03	55.18	52.19	55.07	50.77	55.20	49.71	55.08
6152.00	50.75	37.37	47.97	37.03	51.00	37.55	53.65	36.56
6288.79	93.63	92.66	93.50	92.58	93.66	92.67	93.75	92.58
6289.00	94.14	93.07	93.95	93.02	94.12	93.10	94.30	93.01
7158.63	95.85	94.73	95.55	94.75	95.79	94.78	95.87	94.69
7244.00	96.29	95.37	95.81	95.54	96.12	95.45	96.23	95.48
7705.01	93.97	90.78	92.91	90.98	93.67	90.97	94.37	90.84
8308.00	71.89	62.15	69.60	62.09	71.11	62.68	73.18	61.73
9476.00	77.47	75.43	76.96	75.43	77.52	75.45	78.16	75.51
9478.87	91.82	90.38	91.34	90.47	91.97	90.35	92.16	90.47
9541.41	91.68	89.47	91.04	89.54	91.58	89.56	92.01	89.48
10287.76	78.73	72.57	78.31	71.83	78.87	72.65	79.31	71.66
11109.00	0.08	0.05	0.06	0.05	0.07	0.05	0.09	0.05
